# Common and distinctive genomic features of *Klebsiella pneumoniae* thriving in the natural environment or in clinical settings

**DOI:** 10.1038/s41598-022-14547-6

**Published:** 2022-06-21

**Authors:** Jaqueline Rocha, Isabel Henriques, Margarita Gomila, Célia M. Manaia

**Affiliations:** 1 Universidade Católica Portuguesa, CBQF-Centro de Biotecnologia e Química Fina-Laboratório Associado, Escola Superior de Biotecnologia, Rua Diogo Botelho 1327, 4169-005 Porto, Portugal; 2 Department of Life Sciences, Faculty of Science and Technology, University of Coimbra, Coimbra, Portugal; 3 CESAM, University of Aveiro, Aveiro, Portugal; 4 Microbiologia, Departament de Biologia, Universitat de les Illes Balears, Palma de Mallorca, Spain

**Keywords:** Microbial ecology, Microbiology

## Abstract

The *Klebsiella pneumoniae* complex is comprised of ubiquitous bacteria that can be found in soils, plants or water, and as humans’ opportunistic pathogens. This study aimed at inferring common and distinctive features in clinical and environmental *K. pneumoniae.* Whole genome sequences of members of the *K. pneumoniae* complex (including *K. variicola*, n = 6; and *K. quasipneumoniae*, n = 7), of clinical (n = 78) and environmental (n = 61) origin from 21 countries were accessed from the GenBank. These genomes were compared based on phylogeny, pangenome and selected clinically relevant traits. Phylogenetic analysis based on 2704 genes of the core genome showed close relatedness between clinical and environmental strains, in agreement with the multi-locus sequence typing. Eight out of the 62 sequence types (STs) identified, included both clinical and environmental genomes (ST11, ST14, ST15, ST37, ST45, ST147, ST348, ST437). Pangenome-wide association studies did not evidence significant differences between clinical and environmental genomes. However, the genomes of clinical isolates presented significantly more exclusive genes related to antibiotic resistance/plasmids, while the environmental isolates yielded significantly higher allelic diversity of genes related with functions such as efflux or oxidative stress. The study suggests that *K. pneumoniae* can circulate among the natural environment and clinical settings, probably under distinct adaptation pressures.

## Introduction

The species *Klebsiella pneumoniae*, within the family *Enterobacteriaceae*, is considered a major pathogen, associated with urinary, respiratory, gastrointestinal and skin infections^1,2^. *K. pneumoniae* are severe opportunistic pathogens, whose control is impaired by the frequent multidrug resistance phenotype and genotype, representing a major threat for neonates, elderly, and immunocompromised patients^3^. Previous reports indicate that about one-third of the Gram-negative bacterial infections observed in hospitals are attributed to members of this species^4^. The genome of *K. pneumoniae* is ~ 5.5 Mbp in size, with ~ 5500 genes, of which ~ 3500 are accessory, suggesting its dynamic and plastic character^5,6^. The accessory genome includes acquired antimicrobial and metal resistance and virulence genes, as well as plasmids and other elements associated with horizontal gene transfer, evidenced through functional annotation and comparative genomics and supported by the occurrence of domains of distinct guanine/cytosine content^2,4,7^. A wide range of virulence factors involved in iron uptake, capsule production, biofilm production, and others, facilitate the evasion of host defence mechanisms^8–10^. In addition, members of this species frequently acquire antibiotic resistance genes from all classes of antibiotics, and are among the first to spread emerging resistance genes (e.g., *bla*_CTX_, *bla*_KPC_, *bla*_NDM_)^4,11–13^.

As other opportunistic pathogens, *K. pneumoniae* are ubiquitous bacteria able to thrive in environmental compartments (e.g., soil, plants, and waterways)^1,14^. While some *K. pneumoniae* are likely emitted from human and animal sources, and hence can be considered environmental contaminants, others are truly environmental strains thriving in their natural habitat^15,16^. Indeed, water, vegetation and soil have been described as the native environment of *K.* pneumoniae^17^. And, the sharing of phenotypic features between clinical and environmental isolates of *K. pneumoniae* has been demonstrated^18,19^. However, given its wide distribution^7^, it is important to investigate the common and distinctive genomic features of environmental and human-associated strains of *K. pneumoniae*. Although distinguishing between environmental and human bacteria can be challenging, the characterization of populations from both origins is crucial to assess the major drivers of evolution, the stability of acquired genetic traits, and ultimately to infer about privileged paths of transmission from the environment to humans.

This study aimed to test the hypothesis that human-associated and environmental *K. pneumoniae* may belong to distinct genetic lineages and display different features, particularly regarding acquired genes, allelic genetic diversity, among others. It is also hypothesized that genes acquired by clinical *K. pneumoniae* may be stable in these bacteria when they survive in the environment. The study was designed to cover the broadest geographic distribution of clinical and environmental isolates. Therefore, whole genome sequences of *K. pneumoniae* were retrieved from NCBI database, which were complemented with whole genome sequences available in the in-house collection.

## Results

### Genome’s collection and phylogenetic analysis

The study examined the genomes of 139 isolates, 61 of environmental samples (ENV) and 78 clinical (CLI) (Supplementary Table 1, Supplementary Fig. 1), with origin in 21 countries: USA (23/139, 17%), UK, Portugal and Spain (each 15/139, 33%), China (14/139, 10%), Germany (13/139, 9%), Thailand (11/139, 8%) and other countries (each < 8 isolates, 33/139, 24%). Redundant genomes were excluded by ensuring that each pair sharing 100% of Average Nucleotide Identity (ANI), had origin in a different country or sample type. Although available in the GenBank as *K. pneumoniae*, 13 genomes generated ANIb values of 93–95% with the other 126, which shared among them 98–100%, (Supplementary Table 2). That group of 13 strains was later reclassified by the GenBank *as K. variicola* (n = 6) and *K. quasipneumoniae* (n = 7)^20^. The determination of the *K. pneumoniae* Pasteur multi-locus sequence types (STs) of 139 genomes resulted in 62 STs, 8 of which (ST11, ST14, ST15, ST37, ST45, ST147, ST348 and ST437) included CLI and ENV genomes, 23 STs included only CLI strains and 32 STs only ENV strains (Table 1, Supplementary Table 1, and Supplementary Fig. 2). The predominant STs among CLI genomes were ST147 (18%), ST11, ST23, and ST258 (each 8%) and among ENV genomes were ST14 (8%), ST895 and ST3128 (each 7%) (Table 1 and Supplementary Table 1). Unique STs (n = 36), corresponding to a single genome, were observed in CLI (n = 13) and in ENV (n = 24) isolates in proportions significantly different (Fisher’s Exact test, p < 0.05). Part of these were the genomes latter identified as *K. variicola* (n = 6, 1 CLI and 5 ENV) and as *K. quasipneumoniae* (n = 7, 1 CLI and 6 ENV) and were all unique (Supplementary Fig. 2). The option to maintain the non-*K. pneumoniae* genomes in the study was justified by the fact that they belong to the same species complex and their inclusion avoided the disproportion on the number of CLI and ENV genomes that might bias the results. Possible biases in the results due to the inclusion of these genomes were also critically assessed. The dendrogram representing the ANIb values between pairs of strains clustered the genomes according to the ST although, in some cases, such as the ST11, ST23, ST37, ST258 and ST392, the ST was divided in different groups (Supplementary Fig. 3).

**Table 1:** Summary of the *Klebisella* spp. genomes features used in this study.

	Clinical	Environmental
Number of isolates	78	61
Number of countries	14	14
Sequencing technologies	Illumina PacBio Nanopore	Illumina PacBio 454
Number of STs	31	39
Unidentified STs^a^	No	Yes (n = 1)
**Most abundant STs (identified in at least 3% of the 139 genomes)**		
ST11	6	1
ST14	2	5
ST15	4	2
ST23	6	0
ST37	2	2
ST147	14	2
ST258	6	0
ST307	4	0
ST326	4	0
ST392	4	0
ST895	0	4
ST3128	0	4

^a^Unidentified ST refers to a ST which has not been determined as it has not been possible to obtain the housekeeping gene sequences required for this determination.

**Figure 1:**
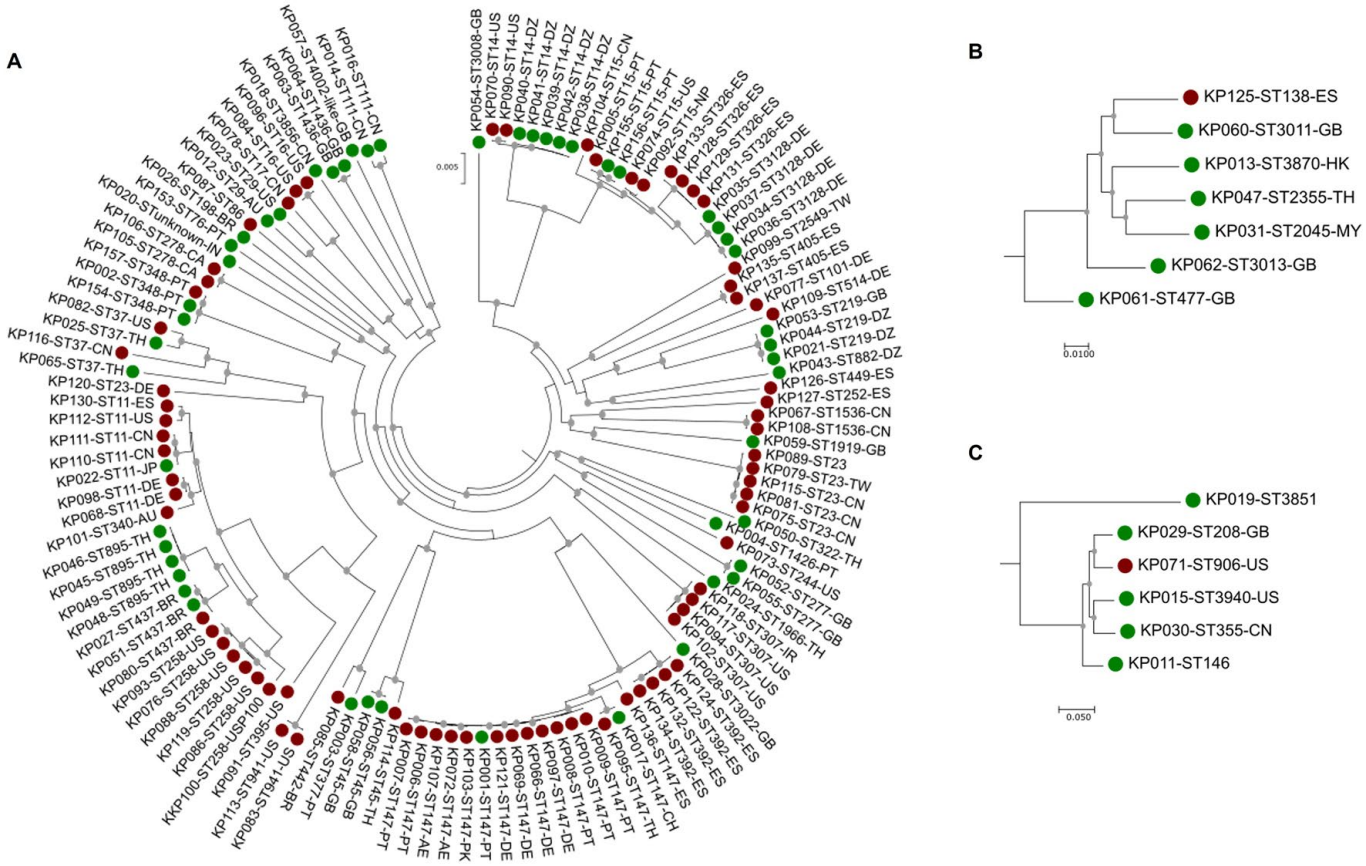
Phylogenetic trees based on the concatenated nucleotide sequences of the 2704 monocopy core genes defined in the (**A**) *K. pneumoniae *sensu stricto (n = 126), (**B**) *K. quasipneumoniae* and (**C**) *K. variicola* genomes analysed. A phylogenetic tree was constructed based on 2,542,200 bp and using the GTR evolutive model, which was determined to be the model that better fitted the data, and (**A**), (**B**) and (**C**) represent zoom sections according to the species. On the labels are indicated the name of the strain genome, the sequence type and the country of isolation. Grey circles in the nodes indicate values of bootstrap above 70%. Red and green circles indicate clinical and environmental genomes, respectively.

Six of the 8 STs that included CLI and ENV genomes, were distributed by different countries. Specifically, ENV isolates with the same ST as the CLI ones were observed in ST11 (1 ENV in Japan and 6 CLI in Germany, China, USA and Spain), ST14 (5 ENV in Algeria and 2 CLI in USA), ST15 (2 ENV in Portugal and 4 CLI in Portugal, Nepal, USA and China), ST37 (2 ENV in Thailand and 2 CLI in USA and China), ST45 (2 ENV in UK and 1 CLI in Thailand) and ST147 (2 ENV in Portugal and Switzerland and 14 CLI in Portugal, Germany, United Arab Emirates, Thailand, Pakistan and Spain) (Supplementary Fig. 2). Also, some STs represented by more than one genome were reported in a single country (USA, ST16, n = 2 CLI; USA, ST941, n = 2 CLI; Portugal, ST348, n = 2 ENV, n = 1 CLI; Spain, ST392, n = 4 CLI; Spain, ST326, n = 4 CLI; Spain, ST405, n = 2 CLI; Germany, ST3128, n = 4 CLI), and most of the times, were reported by the same authors. This latter situation was observed for ST258 in USA (n = 6 CLI) and ST437, (n = 2 ENV, n = 1 CLI) in Brazil (Supplementary Fig. 2). A phylogenetic analysis was performed based on a total of 2704 core monocopy genes of the 139 isolates, relying on the generalized time-reversible (GTR) model, the most suitable to assess the evolution in both CLI and ENV groups (Fig. 1). As expected, the isolates grouped preferentially according to the STs and the core-genome based phylogenetic analysis performed individually for CLI and ENV genomes showed an identical organization (Supplementary Fig. 4). The core-genome based phylogenetic tree showed that in a few cases, isolates of CLI and ENV origins shared high sequence identity of these core genes (100%). This was observed in ST15 and in ST348 in isolates from Portugal, in ST437 in isolates from Brazil, in ST147 in isolates from Portugal (ENV) and Germany (CLI), or Thailand (CLI) and China (ENV), as well as ST11 with isolates from China (CLI) and Japan (ENV) (Fig. 1). An intriguing situation was observed for isolate KP120 whose MLST typing indicated ST23, and the core genome and ANI analysis placed this isolate in a cluster comprised by ST37 isolates. The possibility that is a case of contamination was excluded, since after the first check of genomes, all genomes with > 5% contamination were eliminated from the analyses.

The genomes were further analyzed aiming to infer about features that might be associated to environmental versus clinical origin, species or STs. The scoring of genes in the pan-genome of the 139 genomes^21^ showed that no significant differences of gene annotation and frequency were observed between CLI and ENV genomes. As expected, this analysis revealed the exclusive genes in each of the three species (p < 0.05, Bonferroni), 292 in *K. pneumoniae*, 99 in *K. quasipneumoniae,* and 1638 in *K. varicola* (Supplementary Table 3). Among the exclusive genes in *K. pneumoniae*, more than half (150) were annotated as hypothetical proteins, and others were related with metabolism, virulence and type IV secretion systems, among other (Supplementary Table 4). The comparison of each of the most abundant ST (Table 1) with all the others, did not reveal significant differences in the pangenome composition. Among the STs that included CLI and ENV genomes, only in ST14 isolates were detected gene annotations with significantly different frequency between CLI and ENV genomes (Bonferroni, p < 0.05). A deeper examination of ST14 that comprised 2 CLI and 5 ENV genomes, showed that among the 307 annotations that distinguished both, most (235) were hypothetical proteins, being the remaining related with metabolism, arsenic, chloramphenicol or aminoglycoside resistance, as well as transposons, only present in the CLI genomes (Supplementary Table 4).

### Pangenome and clinical vs. environmental analyses

A similar number of open reading frames (ORFs) was detected in CLI and ENV pangenomes (a total 12,133 genes, 10,210 in CLI and 10,288 in ENV) (Supplementary Fig. 5). The dendrogram representing the matrix of presence/absence of genes and the respective heatmap (Supplementary Fig. 6 and Supplementary Fig. 7) showed that ENV and CLI genomes clustered together. These observations suggest that the pangenome was closely related among members of the same phylogenetic group and geography or CLI *vs*. ENV origin. The CLI and ENV core genes included functional categories mostly related with genetic information processing (587/2713, 21% CLI, 435/2319, 19% ENV), environmental information processing (298/2713, 11% CLI, 261/2319 11% ENV), signalling and cellular processes (298 /2713 11% CLI, 281/2319 12% ENV) and carbohydrate metabolism (288/2713 11% CLI, 249/2319 11% ENV) (Supplementary Fig. 8). In some cases, the array of accessory genes contributed to subdivide a single ST into distinct clusters, suggesting that the profile of gene acquisition was not explained based only on the origin or phylogenetic group. This was observed, for example, for ST11, ST147, ST258, ST392, and ST437 (Supplementary Fig. 7). The absence of significant pan-genome associations with CLI or ENV genomes, motivated a targeted analysis of clinically relevant traits in both groups. Therefore, the genomes were further compared based on presence/absence of alleles of 237 related with antibiotic and metal resistance, plasmid replicon type, virulence, efflux systems, oxidative stress and *quorum sensing*. These 237 genes were screened in the CLI and ENV genomes to estimate prevalence of genes, prevalence of the respective genetic variants (represented by sequences differed in at least one nucleotide) and intra-gene diversity index.

**Figure 2:**
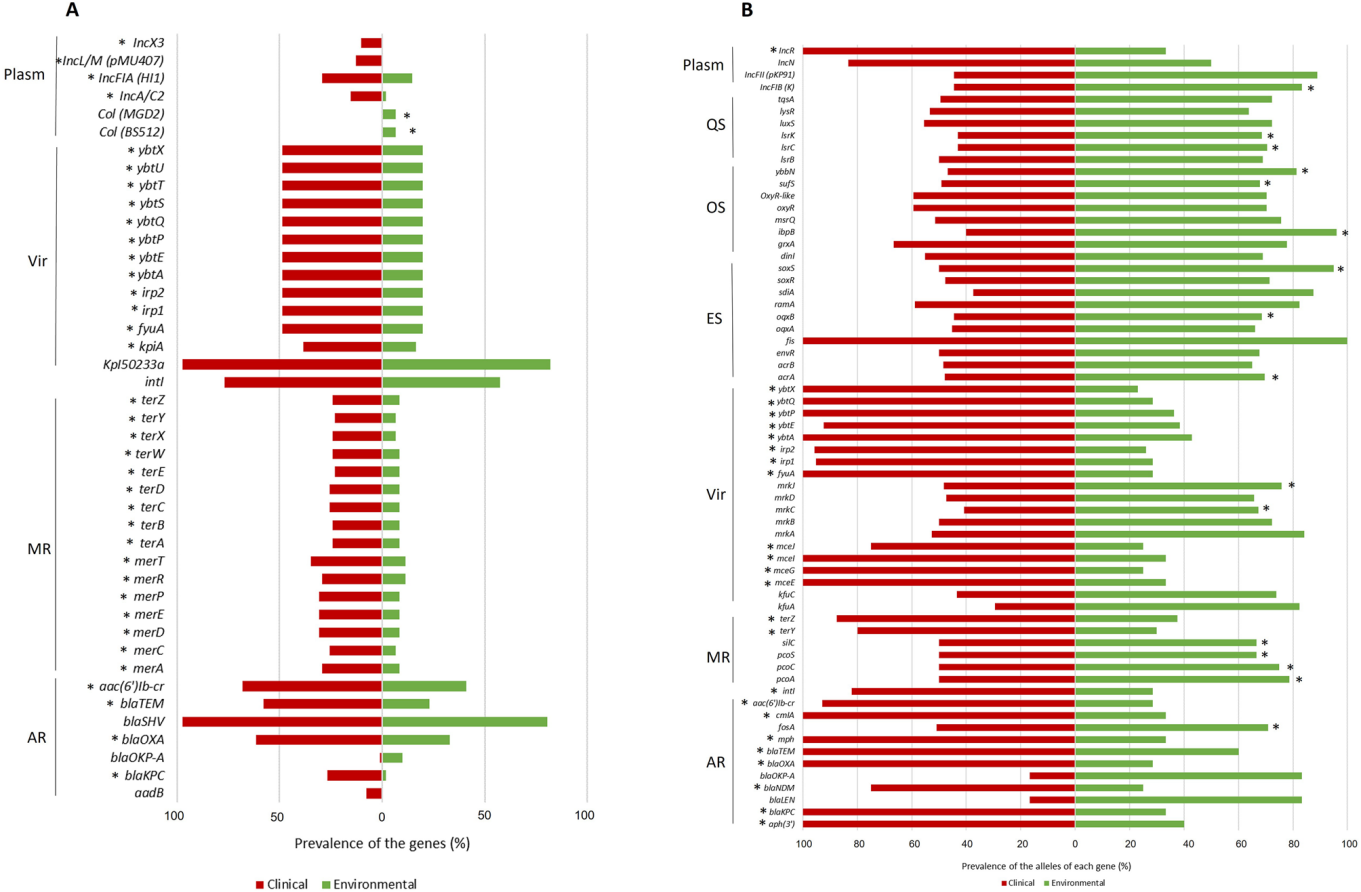
Statistically significant differences observed between clinical and environmental *K. pneumoniae* and closest related species genomes analysed. (**A**) Prevalence (%) of genes (Fisher’s exact test and p-value < 0.05); (**B**) The Shannon diversity index was determined for the alleles of each gene and the genes with significant differences between clinical and environmental genomes were identified (p-value < 0.05). The prevalence (%) of the alleles of these genes, meaning variants of a single gene that differ in at least one nucleotide, is presented. The prevalence of the genes or of the gene-alleles was determined based on the following formula: Prevalence (%) = 100 × (Number of clinical or environmental genomes containing the gene A/total number of clinical or environmental genomes) or 100 × (Number of observed variants of gene A in clinical or environmental genomes/total number of observations of the gene A in clinical or environmental genomes). *Indicates statistically significant differences between clinical and environmental within *K. pneumoniae* genomes. Some genes such as *bla*_LEN_ and *bla*_OKP-A_ were only observed in the *K. variicola* and *K. quasipneumoniae* species, respectively. *AR* antibiotic resistance, *MR* metal resistance, *Vir* virulence, *Plasm* plasmids, *ES* efflux systems, *OS* oxidative stress, *QS* quorum sensing.

Regarding gene prevalence, it was observed that 3 metal resistance (*pbrA, pbrBC* and *pbrR* genes), 37 virulence genes (*iro*, *clb*, *iuc*, among other genes), 13 antibiotic resistance genes (*rmt*, *mef*, *mcr*, among other genes) and 14 plasmid replicon types (*IncHI2*, *IncQ1*, *IncU*, *IncX3*, *psL483*, among others) were detected only in CLI genomes (Supplementary Fig. 9). Only one of these genomes harbouring these exclusive genes, specifically the plasmid replicon type *psL483* was affiliated to *K. quasipneumoniae* (Supplementary Table 5). In contrast, one antibiotic resistance gene and six plasmid replicon types were detected exclusively in ENV genomes (Supplementary Fig. 9). Only one of these genomes was affiliated *K. quasipneumoniae*, the only environmental genome (1/61 genomes) harbouring the replicon types *Col(IMG531)* and *Col(IRGK)* (Supplementary Table 5). These differences apart, 20 genes in these categories were detected in all genomes (antibiotic resistance n = 1, virulence n = 1, efflux systems n = 8, oxidative stress n = 5, *quorum sensing* n = 5) and most genes of the others (162/237) were detected in both groups (Supplementary Table 5 and Supplementary Table 6). However, some of the genes common to both groups presented significantly different prevalence values (p < 0.05). The genes related with antibiotic (*bla*_KPC_, *bla*_OXA_, *bla*_TEM_ and *aac(6′)-Ib-cr*) or metal resistance (*mer*, *ter*) and virulence (yersiniabactin—*fyuA*, *irp*, *ybt*) were significantly more prevalent among CLI genomes, irrespective of the inclusion in the analysis of *K. variicola* strain KP071 (*bla*_OXA_, *bla*_TEM_, *aac(6′)-Ib-cr* and *ter) and K. quasipneumoniae* strain KP125 (*bla*_TEM_) (Fig. 2A). In turn, the resistance genes *bla*_OKP-A_ and replicon types *Col(MGD2)* and *Col(BS512)* were significantly more frequent in ENV genomes (Fig. 2A and Supplementary Table 5). However, this result was attributed to the fact that 6 out of the 7 K*. quasipneumoniae* genomes harboured the gene *bla*_OKP-A_, intrinsic in this species^20^. A dendrogram based on the presence/absence of the 237 genes clustered the genomes in agreement with the sequence types and/or geography and only three genomes, one ENV and two CLI, of distinct ST were identical in this profile (Supplementary Fig. 10). This analysis also showed that a single ST could be subdivided according to geography—e.g. ST14 genomes were divided in Algeria and USA subgroups, a bias that may be related with the distinct pattern of gene association. Also, the ST147 genomes were split into groups from Germany, Portugal, Thailand or United Arab Emirates (Supplementary Fig. 10). Genomes in which was detected the lowest number of the screened genes (< 30% of 237) corresponded to 21 CLI (of 13 STs) and 25 ENV (to 17 STs) genomes. These genomes typically contained metal resistance genes (*pco*, *sil*, *ars*) in ENV genomes, or antibiotic resistance (*bla*_KPC_) and virulence genes (yersiniabactin) in CLI genomes. Genomes with the highest number of the screened genes (> 50% of the 237) corresponded to 3 clinical genomes (ST14, USA and two ST23, China) (Supplementary Table 6).

Regarding the prevalence of alleles of each of the 237 genes, were detected 2661 gene variants (1600 in CLI and 1648 in ENV genomes) (Supplementary Table 7). The rationale of this analysis was to assess genetic variation, irrespective of the implications on the phenotype. The highest number of gene variants was observed for virulence, especially to capsular related genes (*wzc* n = 55, *wzi* n = 64), *quorum sensing* (e.g. *lsrB* n = 74, *tqsA* n = 83) and oxidative stress (e.g. *msrQ* n = 37, *oxyR* n = 37), also observed in *K. quasipneumoniae* and *K. variicola* genomes (Supplementary Table 5). For 29 genes, distributed by all analysed categories, it was observed a statistically significant different (p < 0.05) prevalence allele between CLI and ENV (Supplementary Fig. 9). Twenty six out of those 29 were common to both CLI and ENV, and these were related with antibiotic resistance (n = 4), metal resistance (n = 2), virulence (n = 10), efflux systems (n = 3), oxidative stress (n = 2) and *quorum sensing* (n = 4) and plasmid replicon type (n = 1) (Supplementary Fig. 9). The prevalence of 15 out of these 26 alleles was significantly different, irrespective of the inclusion of *K. quasipneumoniae* and *K. variicola* in the analysis (e.g. *aadA*, *bla*_OXA_, *ibpB*, *terC*, among others). For the other 11 genes out of those 26, *K. quasipneumoniae* and *K. variicola* were responsible for the differences observed between CLI and ENV genomes.

Regarding intra-gene diversity index, for 65 out of the 237 genes, it was observed that CLI and ENV genomes yielded significantly different (p < 0.05) diversity indices (Fig. 2B and Supplementary Table 8). Thirty-nine out the 65 were significantly more diverse among ENV, specifically for antibiotic resistance (n = 3), metal resistance (n = 4), virulence (n = 7), efflux systems (n = 10), oxidative stress (n = 8) and *quorum sensing* (n = 6) and plasmid replicon type (n = 1). Twenty-six out the 65 were significantly more diverse among CLI, specifically for antibiotic resistance (n = 9), metal resistance (n = 2), virulence (n = 12), and plasmid replicon type (n = 3). These differences in diversity indices were maintained when *K. quasipneumoniae* and *K. variicola* genomes were excluded from the analysis, except for 25 genes (Fig. 2B).

Capsular genes are important virulence factors in *Klebsiella* spp., whose products may be determinant for ubiquity and gene flow^10^, justifying the serotyping of the capsular K and lipopolysaccharides O antigens (Supplementary Fig. 11 and Supplementary Table 9). A total of 50 K antigens were detected, being the most predominant the KL64 (13/139), KL2 (8/139), and KL1, KL102, KL15, KL25, and KL62 (each 6/139) (Supplementary Table 9). The antigen types KL64, KL2, KL102, KL15 were detected in CLI and in ENV genomes, although KL64 (12/13) was significantly more frequent in the first (Fisher’s Exact test, p < 0.05). The KL1, reported to be associated to hypervirulent *K. pneumoniae*^22–24^ was only detected in CLI genomes mainly of ST23 (5/6). KL2 antigens also associated with hypervirulent strains^22–24^ were detected in 8 genomes, all ST14 (n = 7) and in ST15 (n = 1). In total, 10 O antigens were detected, being O1/O2v1 (55/139), O1/O2v2 (33/139), O4 (14/139) and OL101 (10/139), the most predominant (Supplementary Table 9). These antigens were detected either in CLI or ENV genomes, although O1/O2v2 was more frequent in clinical genomes (Fisher’s Exact test, p < 0.05).

## Discussion

The hypothesis of the study was that human-associated and environmental *K. pneumoniae* may belong to distinct genetic lineages and yield distinct genome features, mainly those that favour the survival under adverse conditions, such as antibiotic and metal resistance, virulence, oxidative stress or *quorum sensing*^25^. To test this hypothesis, genomes from clinical or environmental sources and wide geographic distribution were compared. In fact, this study revealed that distinct lineages may occur in clinical and environmental settings, although strains closely related to human-associated settings could be detected in the environment. The natural environment may promote the diversification of genetic determinants related with efflux, oxidative stress and *quorum sensing*, while the clinical context may drive the acquisition of virulence, antibiotic and metal resistance genes and plasmid replicon types. A first shortcoming was the limited number of whole genome sequences available for environmental isolates. This suggests the bias existing in public databases towards clinical genomes, which may represent a limitation to investigate the interface between humans and the environment. A solution adopted to overcome this shortcoming was the inclusion of high-quality draft genomes of environmental isolates, which we assumed would support a reliable comparison between CLI and ENV genomes. Also concerning the environmental genomes, the supporting information is sometimes insufficient or inaccurate to support robust ecology studies. The supply of accurate and reliable data relative to environmental isolates should be encouraged among the scientific community.

In the group of genomes that were downloaded for this study, was a group of 13 identified as *K. pneumoniae* and later reclassified as *K. quasipneumoniae* and *K. variicola.* This was also evident in our analysis since these genomes represented distinct groups based on ANIb and phylogenetic analyses. While the whole genome sequence analysis revealed the differentiation of the species, *K. pneumoniae*, *K. quasipneumoniae* and *K. variicola* have been considered phenotypically and phylogenetically close and difficult to distinguish^26^. Nevertheless, it was reported that genes such as *bla*_LEN_, *bla*_OKP_, *gyrA*, *parC*, among others, may contribute to the reliable distinction of these species^20^. In fact, the genes *bla*_OKP-A_ and *bla*_LEN_ were only detected in genomes affiliated to *K. quasipneumoniae* and *K. variicola*, respectively. The gene association analysis of the pangenome confirmed the distinction of the three species. The possible biases due to the inclusion of these 13 genomes (2 clinical and 11 environmental) were identified and discussed. Nevertheless, this situation highlights the importance of the correct species affiliation in public databases, as the information may be used without the verification that was made in this study and that revealed the misidentification. The ST11, ST14, ST15, ST37, ST45, ST147, ST348, ST437 included CLI and ENV genomes, contrasting with the other 54 STs, most represented by a single isolate and a single origin. The STs that we observed to include both CLI and ENV are widely distributed worldwide and have been associated to outbreaks^2^, although do not mirror the predominance of ST258 and ST11 (CG258) reported in representative databases, such as Pathogenwatch (https://pathogen.watch/) (Supplementary Fig. 12). Multidrug resistant isolates are frequently integrated in the CG147, CG15, CG258, combining genes encoding resistance to aminoglycosides, β-lactams, ESBLs, with genetic information associated with extensive lipopolysaccharides diversity^22^. In this study, we observed that genomes affiliated to CG147 (ST147/ST392) mostly yielded KL64 (13/16, 1 ENV and 12 CLIN) while those affiliated to CG15 (ST14/ST15/ST326/ST1328) and to CG258 (ST258/ST11/ST895/ST437) showed higher diversity of capsules antigens (Supplementary Table 7). As described in the literature, from the analysis of genomes yielding KL1 it can be inferred a hypervirulence character. The five genomes with this gene were of CLI origin, belonged to the ST23 and had genes related to yersiniabactin (*ybt*), colibactin (*clb*), aerobactin (*iuc*), salmochelin (*iro*) (Supplementary Table 7). The O1/O2v1 and O1/O2v2 antigens, reported to be associated with hypervirulent strains^22^ were detected both in CLI and ENV genomes, although with higher prevalence in the first group. The relationship between the capsular types and CLI or ENV origin may be also insightful to investigate how this feature may shape the gene flow among *K. pneumoniae* populations, preferentially using a larger sample^10^.

The results of the study show that the environment may represent an important reservoir for *K. pneumoniae* that harbor clinically relevant features. This is illustrated by the lineages closely related with the CG258, ST147, and ST14/15 that included CLI and ENV genomes with wide geographic distribution. While most ENV core genomes were not identical to CLI phylogenetic neighbours, discarding the possibility of clonal spread, in five situations identity values of 100% were observed for the 2704 genes compared (Fig. 1). Remarkably, these situations were noted for isolates affiliated to the CG258 (ST11/ST437), ST147, ST15 and ST348, of high clinical relevance^2,27^, suggesting that the natural environmental can be a suitable habitat for virulent and multidrug resistant strains. However, the results showed that the environment may shape bacterial features. Indeed, the comparison of the pangenome, suggested that the isolates with identical core genomes differed in the accessory genome, hinting the influence of the external factors and eventually of acquired genes (Supplementary Fig. 7).

In order to better explore possible differences between CLI and ENV genomes, we screened 237 genes of clinical relevance and that may be related with the capacity to survive adverse conditions. It was mostly among CLI genomes that exclusive antibiotic and metal resistance, virulence and plasmid replicon types were observed. This observation was not unexpected given the strong selection pressures that bacteria are subjected to in an infection episode^28,29^. While the presence of mobile genetic elements harbouring antibiotic resistance genes may represent a fitness cost in the absence of selective pressures, it will represent an advantage in a clinical context^30^. This may justify the higher diversity observed of genes related to antibiotic, metals, virulence and plasmid replicon types in CLI isolates. Or, in alternative, it may suggest that the presence of such mobile genetic elements contributes to the success of the strains for establishing an infection. Efflux, oxidative stress and *quorum sensing* related genes were common to both groups and in some cases significantly more diverse among environmental genomes. The contribution of the genomes affiliated with *K. quasipneumoniae* and *K. variicola*, mostly of ENV sources, to these results was evidenced, specifically for *envR*, *oxyR*, *luxS*, among others. However, the absence of selection pressure in the environment may be an important driver to promote the diversification of genes and may confer an advantage to adapt to natural environments where external conditions are supposed to vary more than in the human body^31–33^. The continuum between clinical and environmental habitats is known^34–36^ and explains why genes associated to CLI origins (e.g. *bla*_CTX-M_, *bla*_KPC_, *bla*_NDM_) were detected in ENV isolates, although in lower prevalence. In addition, different mobilomes might be associated to CLI or ENV genomes, considering that plasmid replicon types such as *IncA/C2*, *IncFIA(H1)*, *IncL/M (pMU407)*, *IncX3* were more associated to clinical origins and the replicon types *Col(BS512)* and *Col(MGD2)* to the environmental origins. The hypothesis that CLI and ENV genomes yield distinct genome features was confirmed, highlighting what may be the interplay between ecology and evolution.

Metabolism is determinant for the ecology and ubiquity of bacteria and have been demonstrated to be associated with increased virulence and potential to acquire antibiotic resistance in *K. pneumoniae*^37,38^. Blin et al.^39^ observed that the capacity for d-arabinose degradation was associated with hypervirulence in *K. pneumoniae*, a feature that the authors assumed to be linked in the same, acquired, plasmid. Also, Watkins and Maiden^40^ observed that some successful clonal lineages might benefit from allantoin metabolism in the virulent CG23 lineage. These examples suggest that metabolism variation may explain the capability of strains of the *K. pneumoniae* complex to use different carbon, nitrogen and energy sources and inhabit distinct niches. Recently, Hawkey et al.^41^ used a curated collection of *K. pneumoniae* isolates to demonstrate the usefulness of metabolic models to predict phenotype and to reveal strain-specific differences within and between species. In our study, were observed significant differences between ENV and CLI genomes of ST14, some of which were related with metabolism. This result may be influenced by the sample characteristics, as the ST14 isolates were from two countries, the ENV from one and the CLI from another, or by the sample size, as Scoary tool has a higher resolution power for sample sizes larger than 100^21^. Interspecies differences in genes related with metabolism were also observed among *K. pneumoniae, K. varicola* and *K. quasipneumoniae*, which may be related with the higher prevalence among ENV than among CLI of the latter two species. For instance, the genetic pool related with nitrogen sources (N_2_ or fixed sources) has been proposed as an important driver of the ecology of strains of the *K. pneumoniae* complex^39^.

An important conclusion of the study was that distinct lineages prevail in clinical and environmental settings. However, strains closely related with hypervirulent pathogens can be observed in the environment. Another conclusion was that the natural environment may offer favourable conditions for the diversification/evolution of genetic determinants that support adaptation to stress, such as those related with efflux, oxidative stress and *quorum sensing*, while the clinical context seems to drive the acquisition and evolution of virulence and antibiotic and metal resistance genes as well as some plasmid replicon types, which may be selected during colonization or infection. Comparative genomics studies with clinical and environmental isolates will be determinant to better understand the implications and risks of the spread of pathogens in the environment. However, it is essential that the public databases are gradually enriched with complete sequences of environmental isolates, which are still scarce when compared with the data available for clinical isolates.

## Methods

### Whole genome sequences selection

Genomes were searched and retrieved from NCBI, between May 3 and October 31, 2018, using the keyword *Klebsiella pneumoniae*, filtered for assembled genomes (Supplementary Fig. 1). This search resulted in 231 K*. pneumoniae* complete genomes, 174 of clinical origin, 10 environmental and 47 with unreported origin, according to the information available in the NCBI database. For this study, 56 complete clinical genomes were downloaded based on the criterion that they were obtained from different clinical samples (e.g., blood, wound, urine, respiratory tract, among others) and from different countries to avoid the inclusion of repeated isolates. Given the low number of complete genomes of environmental origin, it was necessary to also include 43 draft-genomes available in the same database. Among those of environmental origin (e.g. sewage, river, soil, food) these genomes were selected because they contained fewer contigs or scaffolds (less than 74 and 99, respectively). This collection was complemented with 30 draft genomes (22 clinical and 8 environmental) under study by the same authors^18^ (Gomila et al., in preparation), making a total of 139 genomes, 78 clinical and 61 environmental (Supplementary Table 1). The contamination of the genomes was assessed using the CheckM method, and only genomes with ≤ 5% contamination and ≥ 90% completeness were included in the study^42^. The inclusion of genomes from potentially clonal strains was avoided. For this, when the genomes shared 100% of average nucleotide identity based on BLAST algorithm (ANIb) they were included only when originating from different samples or if they harboured different genes/pangenome.

### Phylogenetic inference

The partial sequences of the housekeeping genes *gapA*, *infB*, *mdh*, *pgi*, *phoE*, *rpoB* and *tonB* were extracted from the genome to determine the Multi-Locus Sequence Type^31^. Sequence types were determined using the BIGSdb^43^. Gene fragments were concatenated and a total of 3012 bp was used for multilocus sequence analysis. Alignment was performed using MEGA7 and phylogenetic trees were constructed using Neighbor-Joining, Maximum Likelihood and Minimum Parsimony methods, with bootstraps of 100 replicates. Average nucleotide identity (ANIb) values (9661 pairwise comparisons) were determined using the online service JSpeciesWS (http://jspecies.ribohost.com/jspeciesws/#analyse). Genomes sharing 100% of ANIb were selected to correspond to different samples in order to avoid repetitions. PRIMER6 software^44^ was used to calculate Euclidean distance and to construct a dendrogram using the Unweighted Pair Group Method using Averages (UPGMA).

### Comparative genomics analysis

All genomes were annotated using PROKKA version 1.12^45^ in order to standardize the annotation for all the genomes. PROKKA software predicts open reading frames and performs the annotation of deduced amino acid sequences. Pangenome analysis of clinical and environmental genomes was determined for each group separately using the GET_HOMOLOGUES software and the criteria 70% similarity and 50% of coverage^46^.

Core genome analysis was performed with three different clustering algorithms, bidirectional best-hits (BDBH), COGtriangle (COG), and OrthoMCL (OMCL). The nucleotide sequences of the monocopy genes from the core genome were aligned using MAFFT v7^47^, and the results were concatenated resulting in alignments of 3,200,905 bp length for clinical isolates, 2,833,245 bp for environmental isolates and 2,542,200 bp for the 139 genomes for subsequent phylogenetic analysis. The best evolutionary model, GTR + I + G + X, was estimated with PartitionFinder 2^48^ using the Bayesian and Beast information criterion. The phylogenetic tree was constructed with PhyML software^49^ and visualized with MEGA7.

To detect sequences exclusive of each group (clinical or environmental) or common to both (139 K*. pneumoniae* isolates), the deduced amino acid sequences belonging to the clinical and to the environmental genomes soft cores were compared using CD_HIT website^50^ (70% similarity over 50% of coverage). The exclusive gene sequences were extracted and validated manually based on BLASTn searches against the opposite group (clinical *vs*. environmental). The functional categories of cloud (genes present in one or 2 genomes), shell (genes present in more than 2 genomes and in less than 95% of the genomes), soft core (genes present in more than 95% of the genomes) and core genes (genes present in more than 99% of the genomes) for all the approaches were determined using the KOALA (KEGG Orthology And Links Annotation) database^51^. Genes encoding putatively clinically- and fitness-relevant properties, such as antibiotic and metal resistance, virulence, *quorum sensing*, and oxidative stress, and sequences of different plasmid replicon types were screened in the whole genome sequences. BLASTn was used to screen genes downloaded from specialized databases. The entire databases were used and a total of 237 genes were detected in at least one genome: metal resistance (n = 38), virulence (n = 87); efflux systems (n = 17) (from Institute Pasteur database, https://bigsdb.pasteur.fr/cgi-bin/bigsdb/bigsdb.pl?db=pubmlst_klebsiella_seqdef&page=downloadAlleles), antibiotic resistance (n = 44) (from ResFinder database^52^ and cross-checked in CARD database, https://card.mcmaster.ca/), plasmids replicon type (n = 35) (PlasmidFinder 2.1 tool from Center for Genomic Epidemiology (https://cge.cbs.dtu.dk/services/PlasmidFinder/), *quorum sensing* (n = 7) and oxidative stress (n = 9) (NCBI). These two last groups were searched in the Uniprot database using the terms “*Klebsiella pneumonia*” and “*quorum sensing*” or “*Klebsiella pneumoniae*” and “oxidative stress” and downloaded from NCBI database. Based on this information, a presence/absence matrix was constructed and similarity between genomes was calculated using the Jaccard index. Results were represented in a UPGMA clustering dendrogram. Variants of each gene were determined based on nucleotide differences (> 1 nucleotide difference) between sequences. The prevalence of the genes or of the gene-alleles was determined based on the following formula: Prevalence (%) = 100 × (Number of clinical or environmental genomes containing the gene A/total number of clinical or environmental genomes) or 100 × (Number of observed variants of gene A in clinical or environmental genomes/total number of observations of the gene A in clinical or environmental genomes).

The capsules and the lipopolysaccharides typing based on K and O antigens was performed using the Kleborate tool^53^.

### Statistical analysis

Fisher’s exact tests were applied to assess the statistically significant differences between the proportion of clinical and environmental isolates that harbored the screened genes and the respective alleles (p < 0.05). The diversity of the alleles observed for each gene was compared based on the Shannon diversity index, which reflects the relative abundance of the gene’s alleles (p < 0.05). Additionally, Roary tool was used to obtain a matrix of gene presence and absence needed as input to the Scoary pipeline^21,54^ which was used to infer possible association between genes and clinical or environmental origins.

## Supplementary Information

Supplementary Figures.

Supplementary Information 1.

Supplementary Information 2.

## Data Availability

The genomes analysed during the current study are available in the NCBI/GenBank repository. The links to the genomes analysed are available in the Supplementary Table 1.
