# Supplementary material for: Common and distinctive genomic features of Klebsiella pneumoniae thriving in the natural environment or in clinical settings
Source: Sci Rep. 2022 Jun 21;12:10441. doi: 10.1038/s41598-022-14547-6 (PMC9213442; doi:10.1038/s41598-022-14547-6)
Supplement: Supplementary file 1 — Supplementary Figures. [file 41598_2022_14547_MOESM1_ESM.pdf]

Supplementary Figures

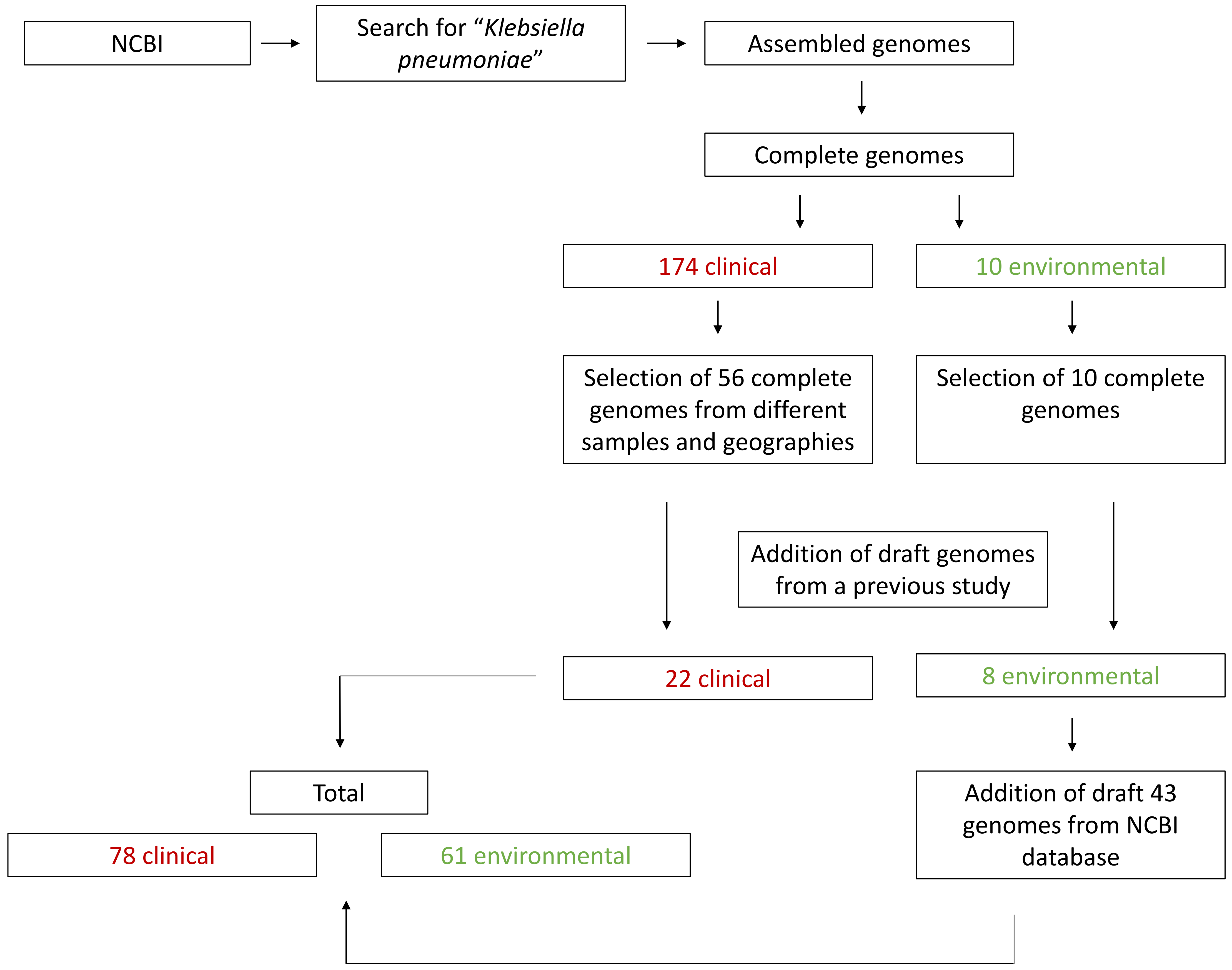

Supplementary Fig. 1 – Workflow followed to establish the *K. pneumoniae* and closest related species colletion of genomes used in this study.

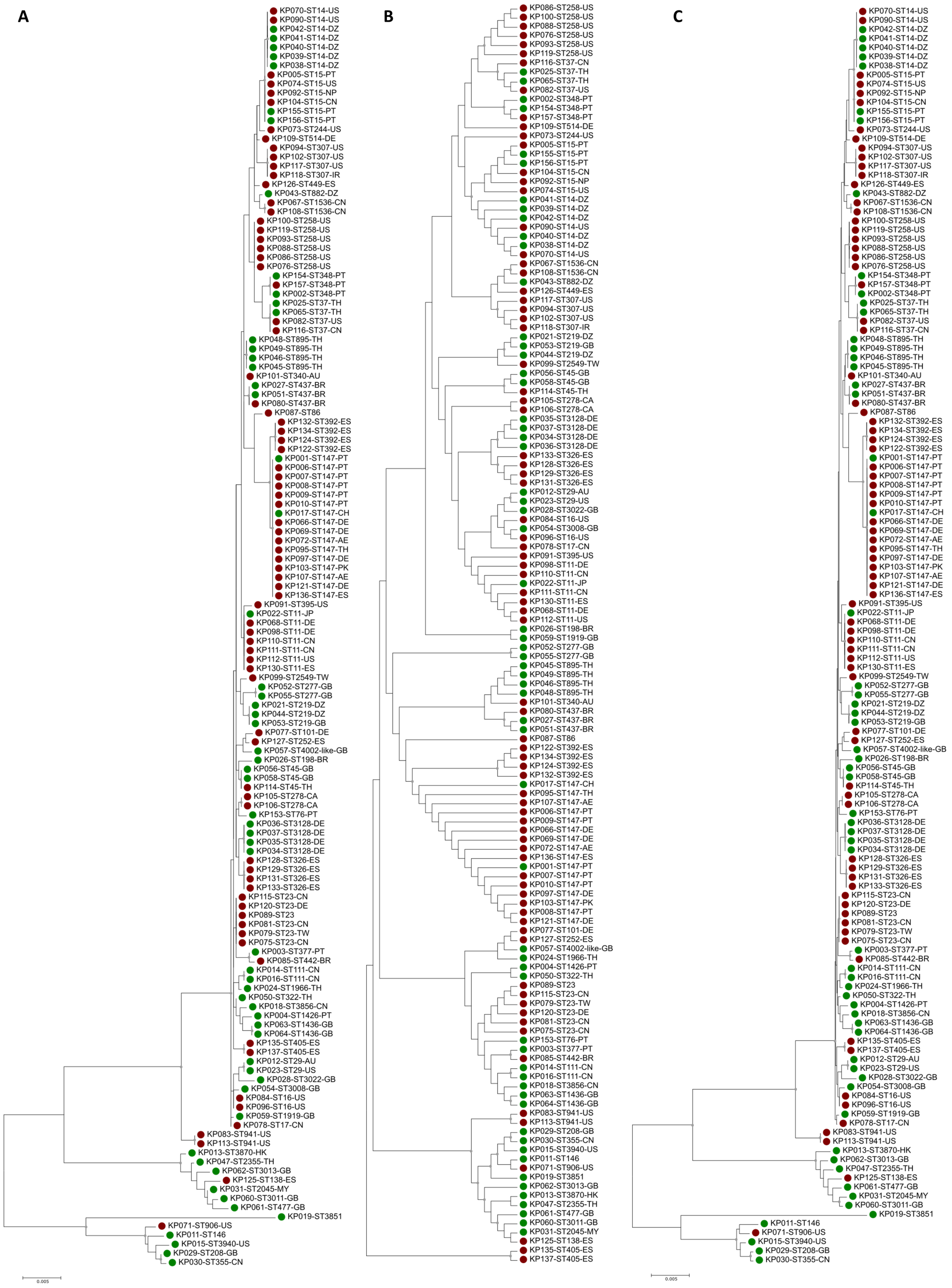

Supplementary Fig. 2 - Phylogenetic tree obtained concatenating MLST gene sequences (*gapA*; *infB*; *mdh*; *pgi*; *phoE*; *rpoB*; *tonB*) of *K. pneumoniae* and closest related species genomes analysed using the Maximum Likelihood (A), Maximum Parsimony (B) and Neighbor-Joining methods. Grey circles in the nodes indicate values of bootstrap above 60%. Red and green circles indicate clinical and environmental genomes, respectively.

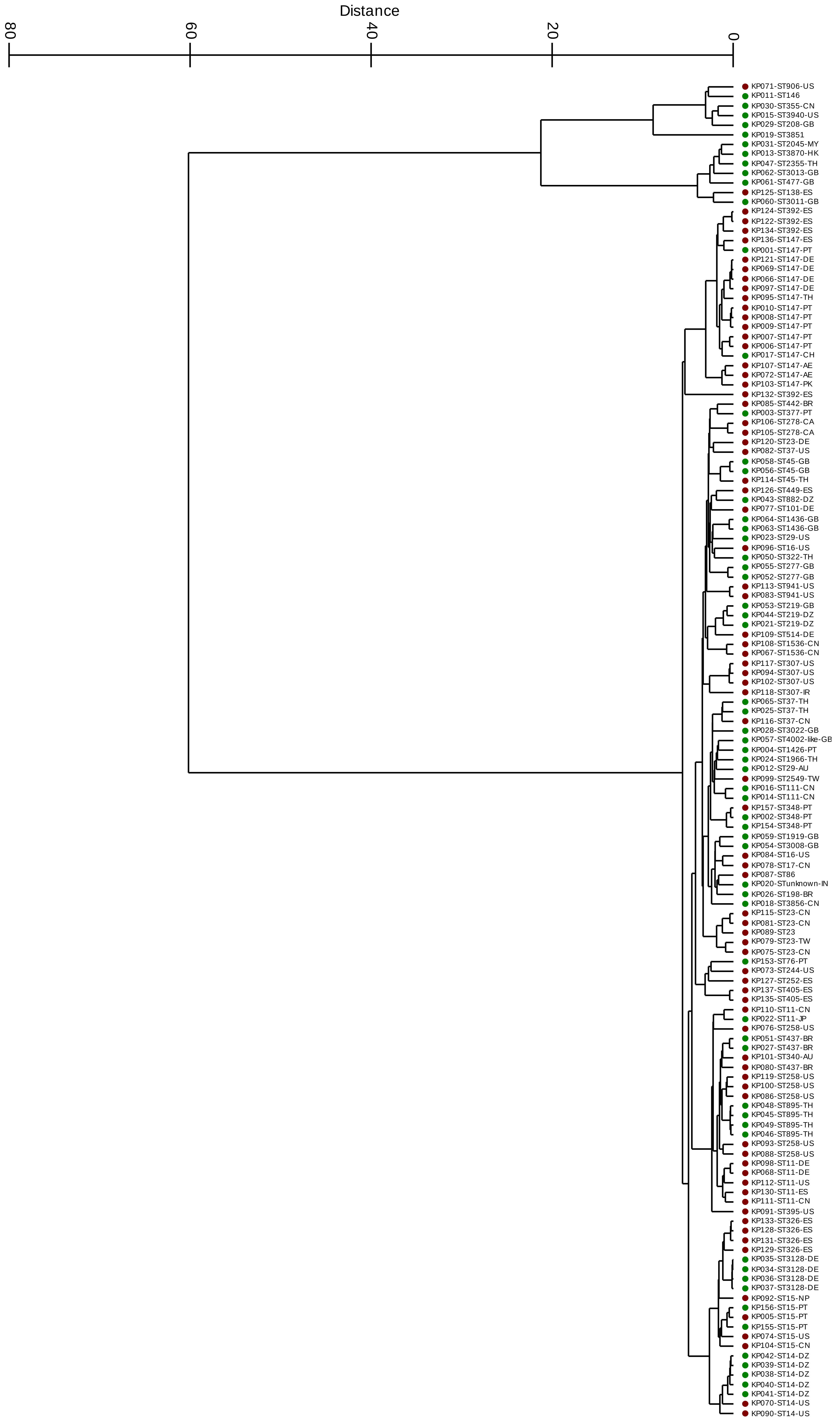

Supplementary Fig. 3 – UPGMA dendrogram based on pairwise ANIb comparisons among the 139 genomes of *K. pneumoniae* and closest related species analysed. Red and green circles represent clinical (n=78) and environmental (n=61) genomes analysed, respectively.

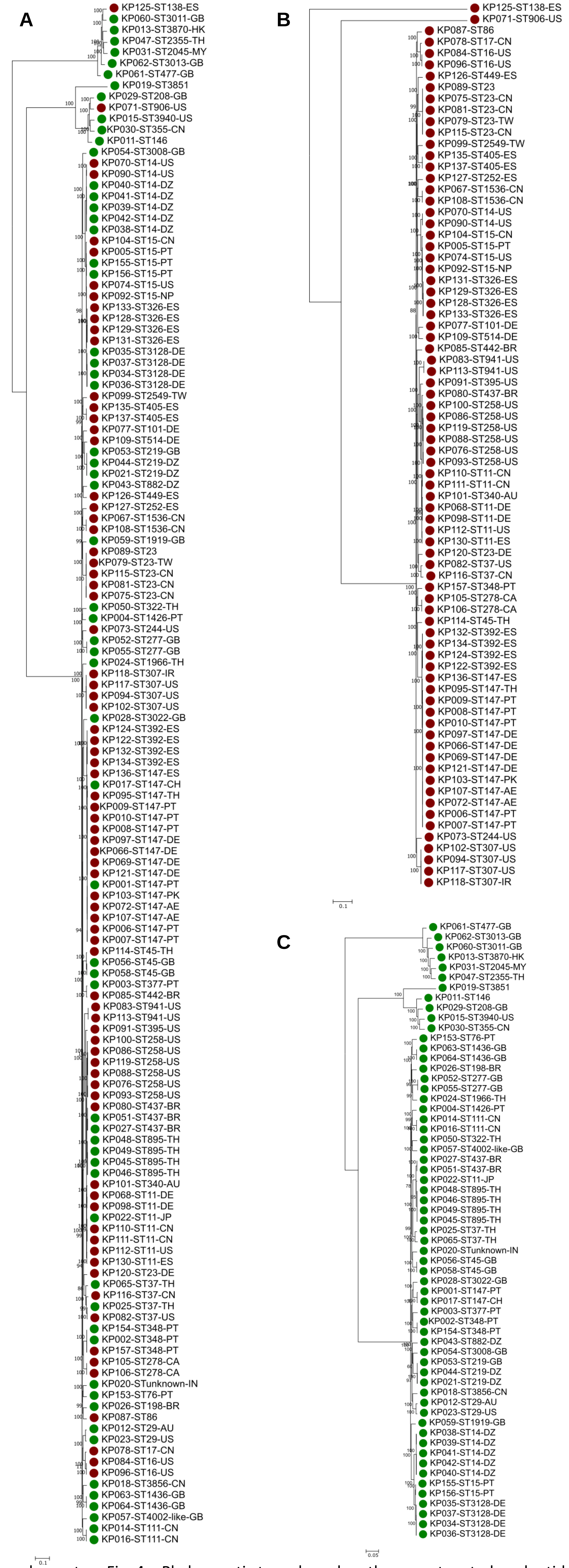

Supplementary Fig. 4 – Phylogenetic trees based on the concatenated nucleotide sequences of A) the 2704 monocopy core genes (2,542,200 bp) defined in all the genomes analysed, B) the 3403 monocopy core genes (3,200,905 bp) defined in the clinical genomes, and C) the 3007 monocopy core genes (2,833,245 bp) defined in the environmental genomes analysed. These phylogenetic tree was constructed using the GTR evolutive model, which was determined to be the model that better fitted the data. On the labels are indicated the name of the strain genome, the sequence type and the country of isolation. Bootstrap values higher than 60% are indicated at the nodes. Red and green circles indicate clinical and environmental isolates, respectively.

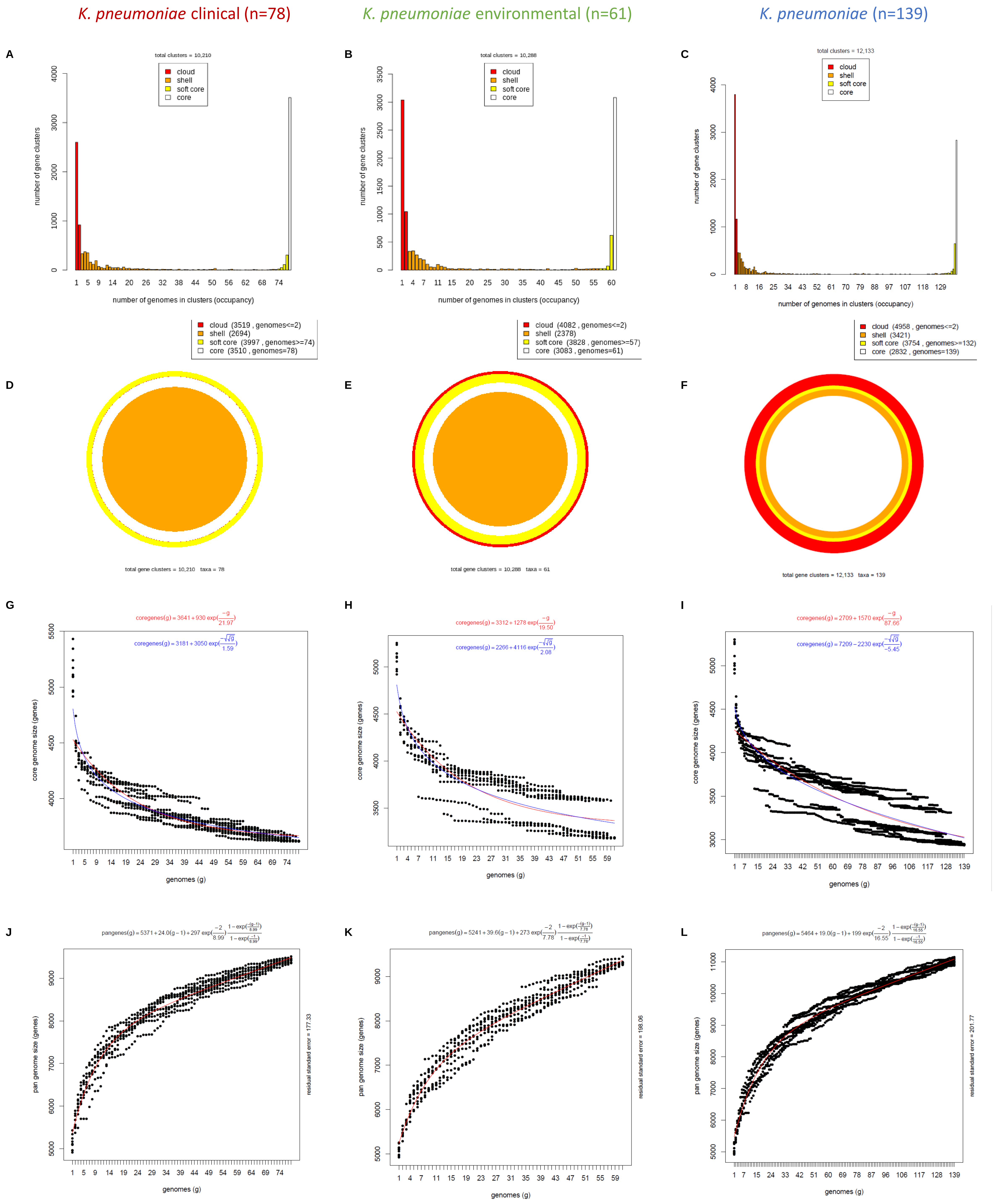

Supplementary Fig. 5– Core and pangenome analysis of clinical, environmental and *K. pneumoniae* and closest related species genomes analysed based on the criteria of 50% of coverage and 70% of similarity between amino acid sequences. A-F) Partition of the OMCL pangenomic matrix into shell, cloud, soft-core, and core compartments created with GET_HOMOLOGUES. Core genes – present in all genomes; soft core genes – present in 95% of the genomes, cloud genes – present 1 or 2 genomes, and shell genes – present in more than 2 and less than 95% of the genomes. G-I) Estimative of the core genome size with the BDBH algorithm. J-L) Estimative of the pangenome size with the BDBH algorithm.

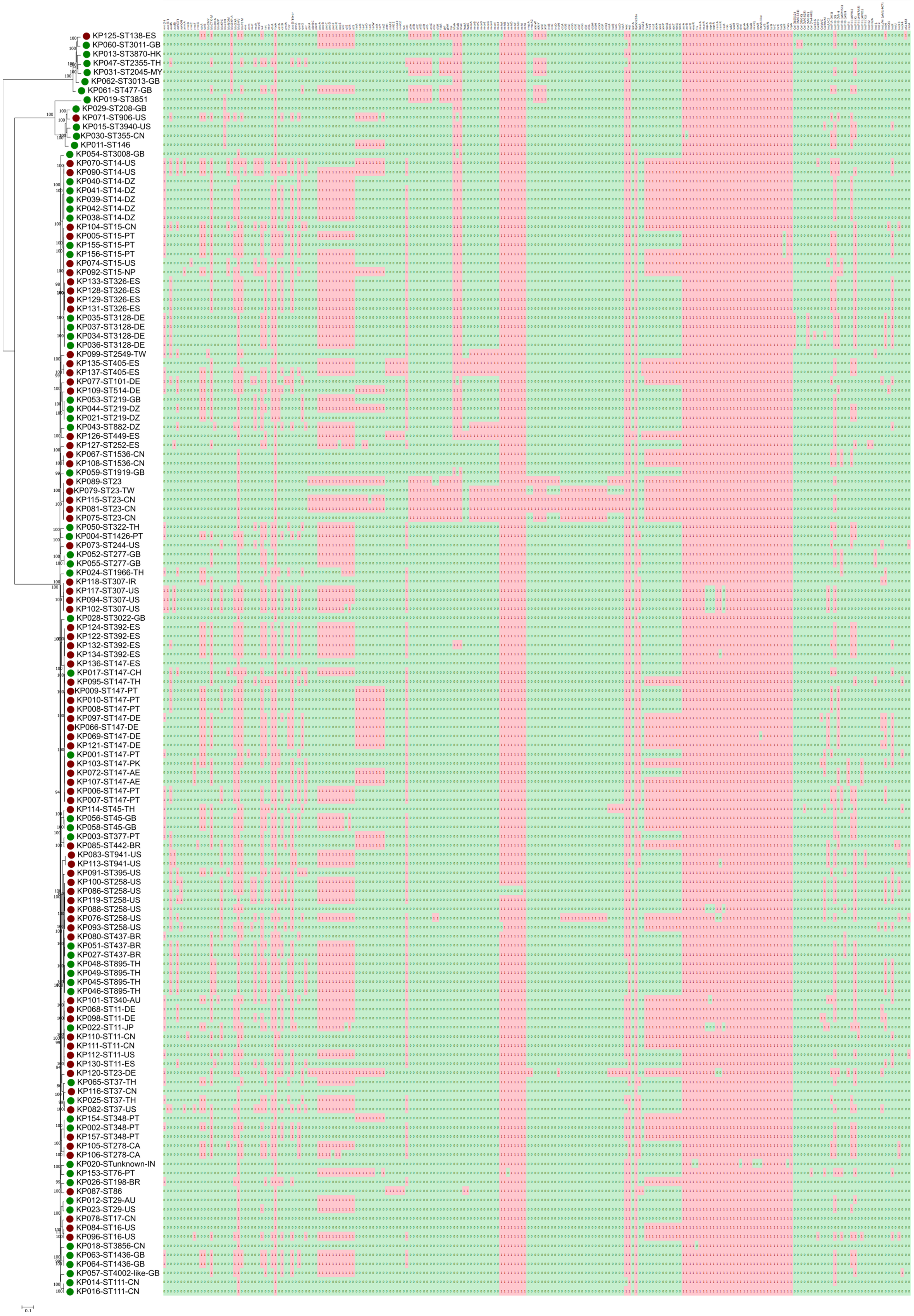

Supplementary Fig. 6 – Phylogenetic tree based on the concatenated nucleotide sequences of the 2704 monocopy core genes (2,542,200 bp) defined in all the genomes analysed. This phylogenetic tree was constructed using the GTR evolutive model, which was determined to be the model that better fitted the data. A matrix based on clinically relevant genes search was coupled to the phylogenetic tree. In this matrix gene presence is presented in red and gene abscence is presented in green. On the labels are indicated the name of the strain genome, the sequence type and the country of isolation. Bootstrap values higher than 60% are indicated at the nodes. Red and green circles indicate clinical and environmental isolates, respectively.

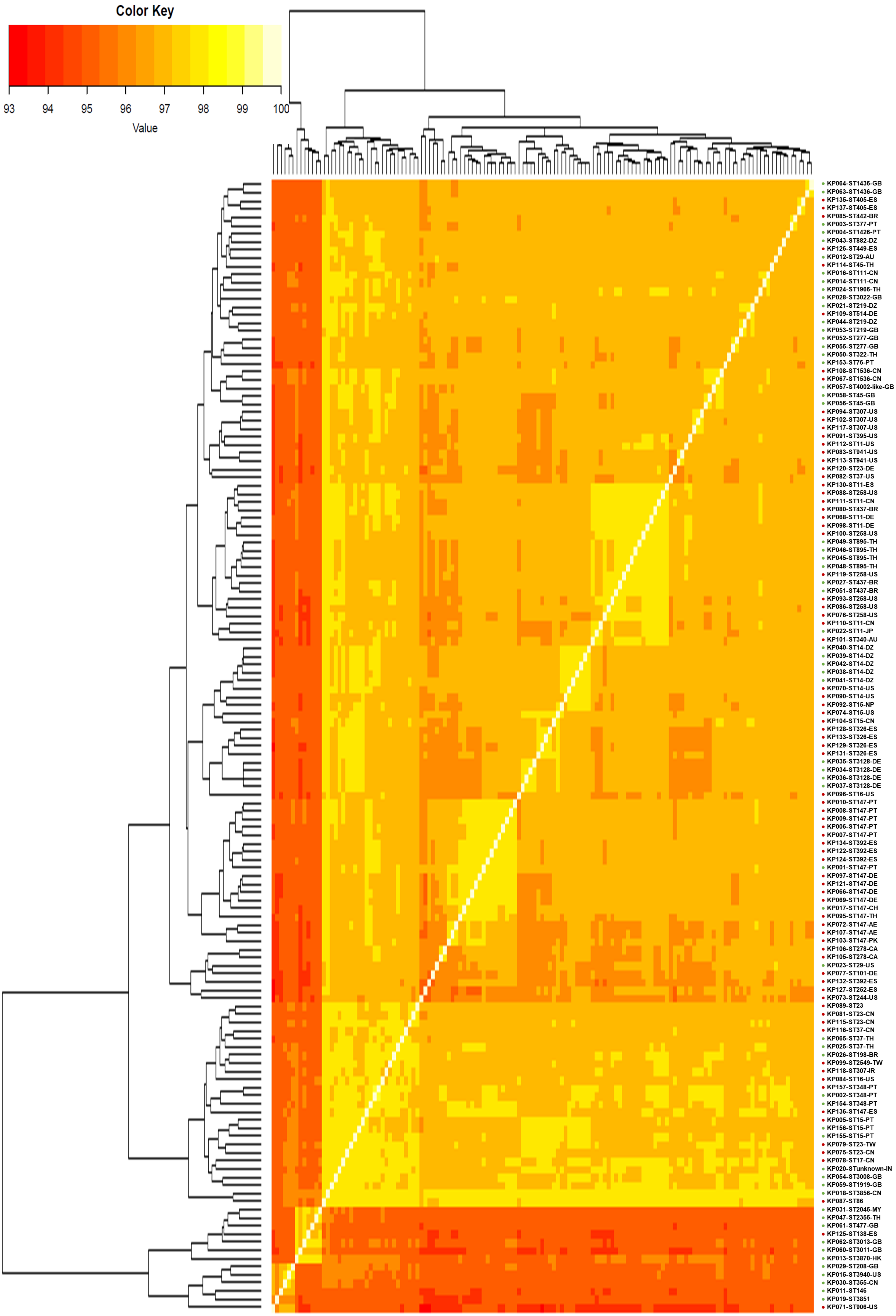

Supplementary Fig. 7 – Heatmap representing the degree of similarity on the genomes based on the presence and absence of protein coding genes (n=12133) in the the *K. pneumoniae* and closest related species genomes analysed. High similarities are indicated in yellow and low similarities in red.

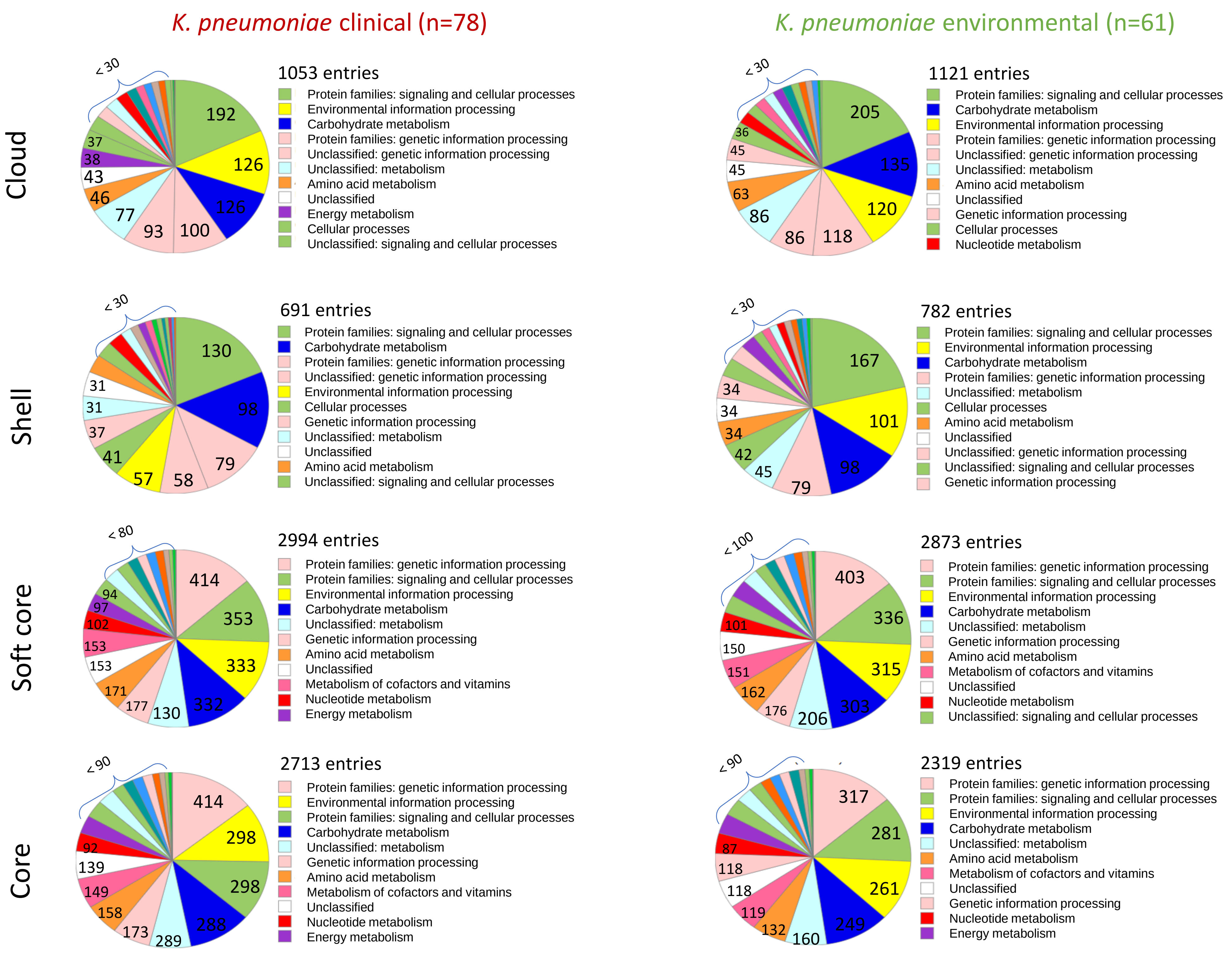

Supplementary Fig. 8 – Functional categories of the amino acid sequences obtained in the pangenome analysis based on the clinical and environmental *K. pneumoniae* and closest related species analysed. Metabolism functional category include the subcategories carbohydrate, energy, lipid, nucleotide, amino acid metabolismo, glycan biosynthesis, metabolism of cofactors and vitamins, metabolism of terpenoids and polyketides, biosynthesis of other secondary metabolites, and xenobiotics biodegradation and metabolism. Genetic information processing functional category include the subcategories translation, folding, sorting and degradation, and replication and repair. Environmental information processing functional category include the subcategories membrane transport, and signal transduction. Cellular processes functional category include the subcategories transport and catabolism, cell growth and death, cellular community – prokaryotes, Cell motility[51].

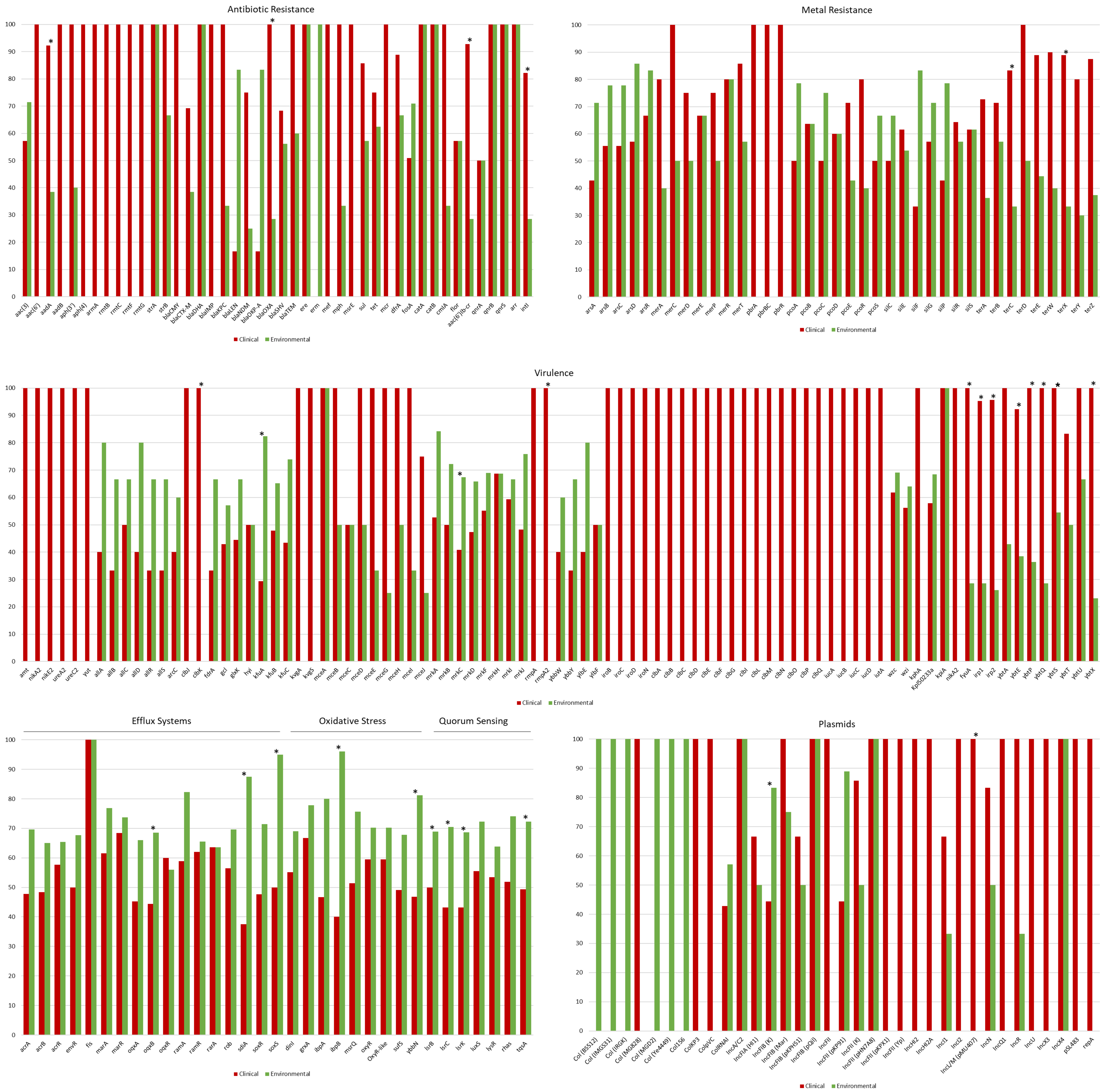

Supplementary Fig. 9 – Prevalence in percentage of the alleles found for each gene in clinical (n=78) and environmental (n=61) genomes analysed. The genes were organized by functional category. The asterisk indicates statistically significant differences between the prevalence of alleles found in the total of clinical and the total of environmental isolates compared to the total alleles found for the same gene, based on Fisher's exact test and p-value < 0.05.

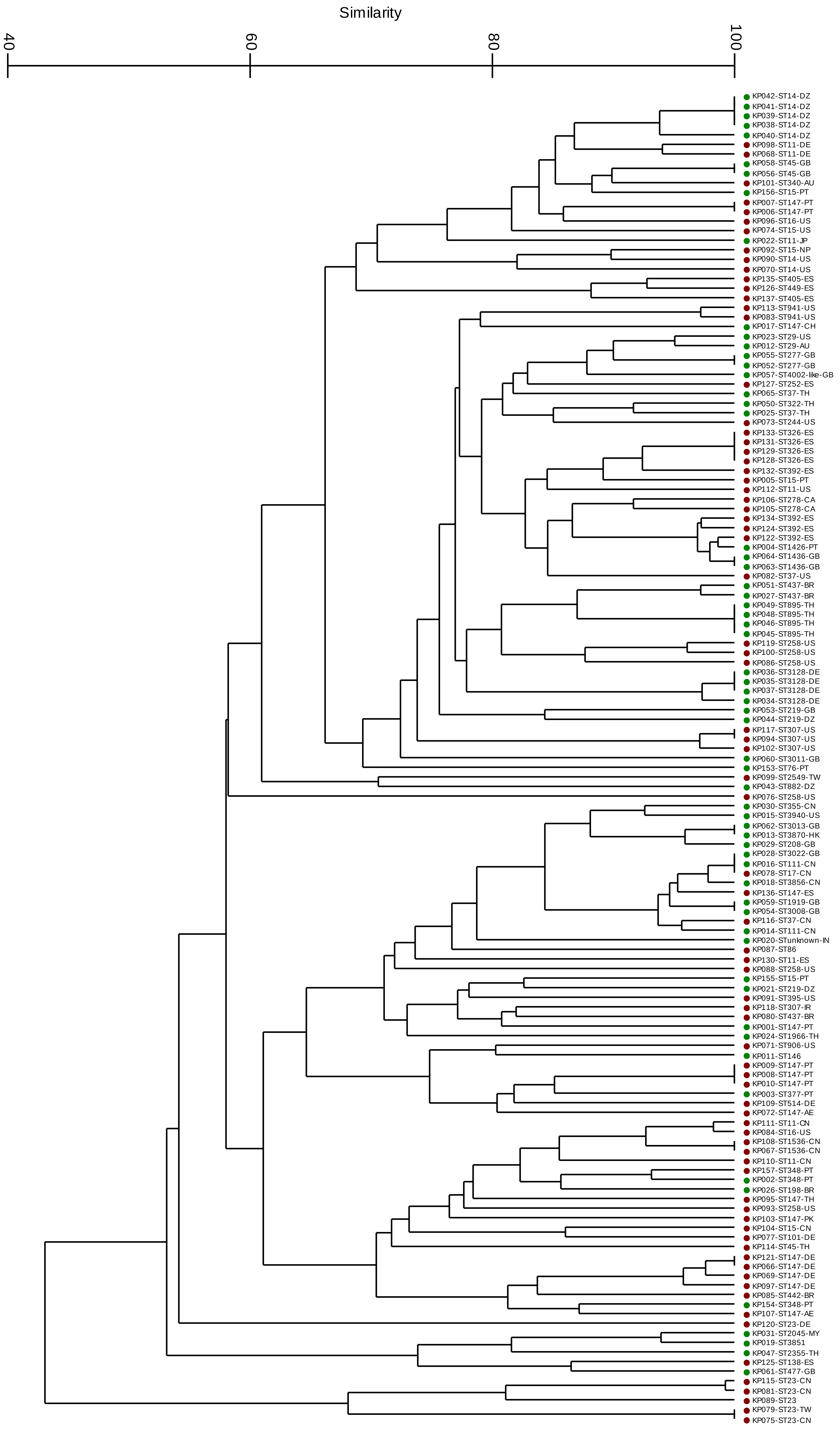

Supplementary Fig. 10 – UPGMA dendrogram obtained from a matrix based on presence and absence of clinically relevant genes in the 139 genomes of *K. pneumoniae* and closest related species analysed. The clustering method was the Jaccard index. Red and green circles represent clinical (n=78) and environmental (n=61) genomes analysed, respectively.

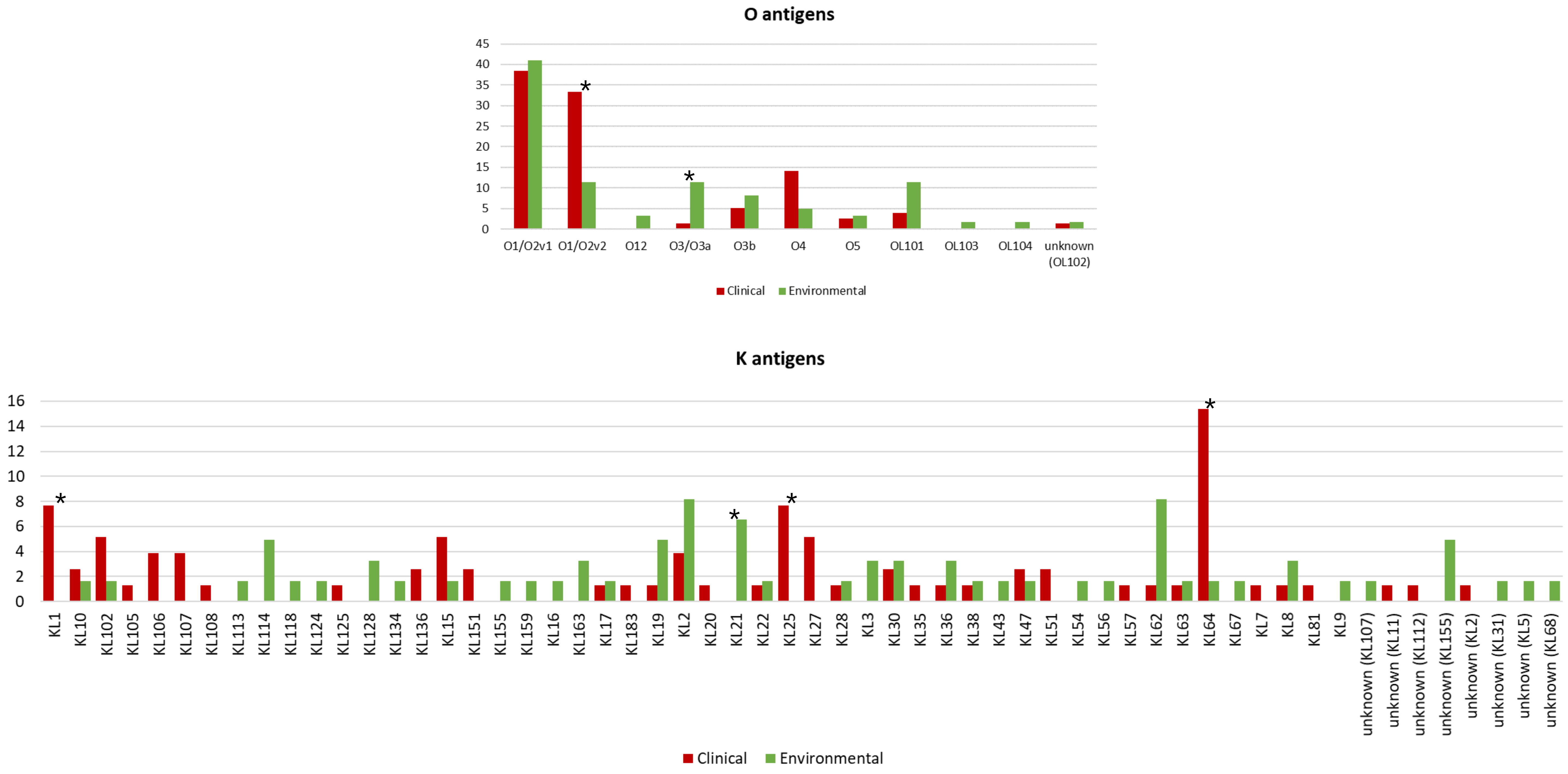

Supplementary Fig. 11 – Prevalence in percentage of the lipopolysaccharides (O antigens) and capsules (K antigens) antigens for clinical (n=78) and environmental (n=61) genomes analysed. The asterisk indicates statistically significant differences between the prevalence of the antigen found in the clinical and in the environmental genomes compared to the total number of clinical and environmental genomes, respectively, based on Fisher's exact test and p-value < 0.05.

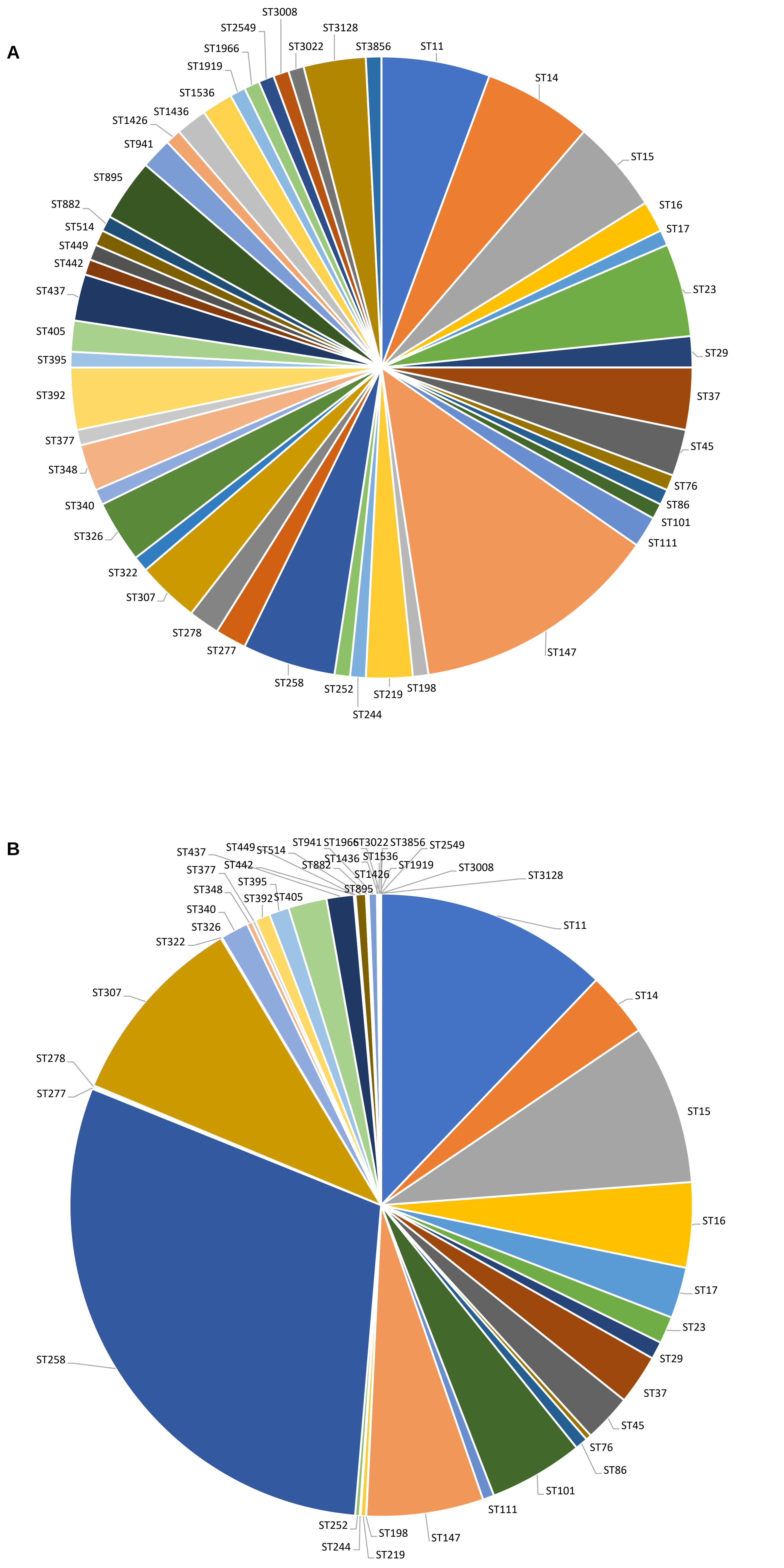

Supplementary Fig. 12 – Graphical representation of the proportion of the sequence types A) detected in the genomes of *K. pneumoniae* used in this study and B) the number of genomes of the same sequence type deposited in the Pathogenwatch database (https://pathogen.watch/) accessed on the 28/03/2022.
